# Multi-ancestry analysis of *POLG* variants in Parkinson’s disease

**DOI:** 10.64898/2026.06.07.26354811

**Published:** 2026-06-08

**Authors:** Yi Wen Tay, Inas Elsayed, Dennis Yeow, Mikayla James, Pin-Jui Kung, Laurel Screven, Allison A. Dilliott, Roy N. Alcalay, Zih-Hua Fang, Ai Huey Tan, the Global Parkinson’s Genetics Program (GP2), Carolyn M Sue, Lara M Lange, Maria Teresa Periñan

**Affiliations:** 1 Department of Biomedical Science, Faculty of Medicine, University of Malaya, Kuala Lumpur, Malaysia; 2 Faculty of Pharmacy, University of Gezira, Wad Medani, Sudan; 3 Systems Biology Ireland, School of Medicine and Medical Science, University College Dublin, Dublin, Ireland; 4 Neuroscience Research Australia, Randwick, New South Wales, Australia; 5 Translational Neurogenomics Group, Garvan Institute of Medical Research, Sydney, NSW, Australia; 6 Molecular Medicine Laboratory and Neurology Department, Concord Repatriation General Hospital, Concord, NSW, Australia; 7 Faculty of Medicine and Health, University of Sydney, Camperdown, Australia; 8 Center for Alzheimer’s and Related Dementias (CARD), National Institute on Aging and National Institute of Neurological Disorders and Stroke, National Institutes of Health, Bethesda, MD, USA; 9 Division of Plastic Surgery, Department of Surgery, National Taiwan University Hospital, Taipei, Taiwan; 10 The Global Parkinson’s Genetics Program (GP2), Bethesda, USA; 11 Parkinson’s Foundation, NewYork, NY 10018, USA; 12 Neurological Institute, Tel Aviv Sourasky Medical Center, Tel Aviv, Israel; 13 Department of Neurology, Columbia University Irving Medical Center, NYC, NY 10032, USA; 14 DataTecnica LLC, Washington, DC 20037, USA; 15 Division of Neurology, Department of Medicine, and The Mah Pooi Soo & Tan Chin Nam Centre for Parkinson’s & Related Disorders, Faculty of Medicine, University of Malaya, Kuala Lumpur, Malaysia; 16 Faculty of Medicine, University of New South Wales, Sydney, NSW, Australia; 177 Department of Neurology, South Eastern Sydney Local Health District, Sydney, NSW, Australia; 18 Laboratory of Neurogenetics, National Institute on Aging, National Institutes of Health, Bethesda, MD, USA; 19 Institute of Neurogenetics, University of Luebeck, Luebeck, Germany; 20 Centre for Preventive Neurology, Wolfson Institute of Population Health, Queen Mary University of London, London, United Kingdom; 21 Unidad de Trastornos del Movimiento, Servicio de Neurología y Neurofisiología Clínica, Instituto de Biomedicina de Sevilla, Hospital Universitario Virgen del Rocío/Consejo Superior de Investigaciones Científicas (CSIC)/Universidad de Sevilla, Seville, Spain

**Keywords:** *POLG*, Parkinson’s disease, genetics, multi-ancestry analysis

## Abstract

**Introduction:**

Variants in the *polymerase gamma* (*POLG)* gene are associated with a wide range of mitochondrial disorders. Emerging evidence suggests a potential link between *POLG* variants and Parkinson’s disease (PD); yet, results remain inconclusive.

**Objectives:**

To investigate the genetic spectrum and prevalence of *POLG* variants in PD across diverse ancestries.

**Methods:**

We leveraged multi-ancestry genetic data from the Global Parkinson’s Genetics Program (GP2), including genotyping data from 98,589 and short-read sequencing data from 36,022 individuals. We performed a *POLG* rare variant screen, case-control association, and gene-level burden analyses.

**Results:**

Five PD cases carried potentially biallelic rare pathogenic/likely pathogenic *POLG* variants. Additionally, 228 individuals (<1%; 161 PD cases, 28 individuals with other neurological disorders, and 39 controls) carried 34 distinct rare pathogenic/likely pathogenic heterozygous variants, with no significant frequency differences between cases and controls, except for the p.Ala467Thr variant in the European population. The co-inherited pathogenic variants p.Thr251Ile and p.Pro587Leu were present in <1% of both cases and controls, with no significant group differences. Burden and variant-level association analyses showed no association between rare *POLG* variant burden or common *POLG* variant enrichment and PD.

**Conclusions:**

*POLG* variants are overall rare in PD. The identification of rare pathogenic variants among PD cases suggests that *POLG*-related mitochondrial dysfunction may contribute to PD in isolated instances, particularly under recessive inheritance. Our findings support a role for *POLG* variants in select cases and underscore the need for larger-scale sequencing and functional studies.

## Introduction

The *polymerase gamma* (*POLG)* gene encodes the catalytic subunit of mitochondrial DNA (mtDNA) polymerase, which is essential for mtDNA replication and maintenance^1^. Genetic variants in *POLG* have been associated with mtDNA depletion or deletion, contributing to various mitochondrial disorders, including progressive external ophthalmoplegia (PEO), Alpers syndrome, SANDO (sensory ataxia, neuropathy, deafness, and ophthalmoplegia), and other mitochondrial recessive ataxia syndromes.^1,2^ To date, more than 300 pathogenic *POLG* variants have been reported, most of which have been identified with an autosomal recessive inheritance pattern, whereas fewer exhibit autosomal dominant inheritance.

Beyond its established role in mitochondrial disorders, emerging evidence suggests a potential link between *POLG* and Parkinson’s disease (PD), possibly through impaired mtDNA maintenance leading to impaired mitochondrial function, increased oxidative stress and neuronal loss in the substantia nigra.^3^ Postmortem brain analyses have revealed high levels of mtDNA deletions in dopaminergic neurons of the substantia nigra in both PD patients and aged individuals.^4–6^ In mouse models, intracerebral injection of damaged mtDNA induced PD-like symptoms that propagated in a prion-like manner.^6^ Moreover, patients with *POLG*-related mitochondrial disease exhibit marked neuronal cell loss in the substantia nigra, with α-synuclein pathology detected in some cases, further implicating *POLG*-mediated mitochondrial dysfunction in neurodegeneration.^7^

Genetically, both monoallelic and biallelic rare pathogenic *POLG* variants have been identified in cases with early- and late-onset PD and parkinsonism, sometimes accompanied by additional neuromuscular or peripheral features such as external ophthalmoplegia or sensory neuropathy.^8–14^ In families with PEO, *POLG* pathogenic variants have also been found to co-segregate with PD symptoms in multiple affected family members.^3^ Case-control association studies have investigated the potential association between more common *POLG* variants and PD risk, generally showing no significant enrichment in cases compared to controls.^15,16^ In contrast, CAG repeat expansions in exon 2 of *POLG* have been linked to increased risk of PD in Finnish, Norwegian, North American, Swedish, and Chinese populations.^15,17–21^ However, conflicting results have also been reported,^22^ leaving the relationship between *POLG* variants and PD risk unresolved.

In this study, we aimed to investigate the frequency and genetic spectrum of rare pathogenic and common *POLG* variants in individuals with PD, atypical parkinsonism, and healthy controls across diverse ancestries. We addressed this by leveraging large-scale, multi-ancestry datasets generated within the Global Parkinson’s Genetics Program (GP2), including NeuroBooster Array (NBA) genotyping, short-read whole-genome sequencing (WGS), and clinical exome sequencing (CES).

## Methods

### Demographics

The demographics of the study cohort are detailed in Supplementary Table 1. We analyzed raw and imputed NBA genotyping data from GP2 Data Release 11 (https://gp2.org/; DOI 10.5281/zenodo.17753486), comprising a total of 98,589 samples: 52,257 PD cases, 5,580 individuals with other phenotypes (atypical parkinsonism and other neurodegenerative conditions), 39,470 healthy controls, and 1,282 unaffected family members of PD cases.

We further utilized the WGS dataset from GP2 Data Release 11, including 28,190 samples: 15,483 PD cases, 3,974 individuals with other phenotypes, 7,585 healthy controls, and 1,148 unaffected family members of PD cases. In addition, we incorporated CES data from 7,832 PD cases included in GP2 Data Release 11, generated by the Parkinson’s Foundation (PD GENEration), a large multi-center initiative based in North America.^23^ Individual cohort recruitment, sample collection, and data collection within GP2 have been described previously.^24–26^

All samples underwent genetic ancestry predictions using the GenoTools pipeline (https://github.com/GP2code/GenoTools), as previously described.^27^ Individuals were classified into eleven genetic ancestry groups: African Admixed (AAC), African (AFR), Ashkenazi Jews (AJ), Latinos and Indigenous People of the Americas (AMR), Complex Admixture History (CAH), Central Asian (CAS), East Asian (EAS), European (EUR), Finnish (FIN), Middle Eastern (MDE), and South Asian (SAS).

### Quality Control

For the NBA genotyping data, PLINK binary files were processed using the GenoTools pipeline, following standardized quality control (QC) procedures described previously.^27^ Briefly, samples were excluded if they failed any of the following criteria: call rate < 0.95, inconsistency between genetically inferred and reported sex, or excess heterozygosity (|F|>0.15). After preliminary sample-level QC, variants with Hardy-Weinberg Equilibrium (HWE) ≤1×10^−5^ in control samples were removed. Variants showing non-random missingness by case-control status (P≤1×10^−4^) or by haplotype (P≤1×10^−4^) were also excluded.

For sequencing data, PLINK binary files were processed using the same GenoTools pipeline, following standardized QC procedures as described above. High-quality variants were retained using the following thresholds: call rate >0.95, genotype quality >20, read depth >5, and heterozygous allele balance between 0.25 and 0.75.

### *POLG* rare variant screening

*POLG* genomic coordinates were obtained from the Ensembl database (hg38: chr15: 89,305,198–89,334,861; https://www.ensembl.org).

For the WGS and CES datasets, *POLG* variants were extracted from variant call format (VCF) files using Bcftools (http://samtools.github.io/bcftools/howtos/publications.html) and annotated with ANNOVAR^28^ and Slivar (https://github.com/brentp/slivar). Slivar-specific parameters were used to filter variants by population allele frequencies, and rare variants with a gnomAD PopMax allele frequency <0.005 were retained. To enhance clinical relevance, only coding variants predicted to affect protein function were considered. The known co-inherited *POLG* variants p.Thr251Ile and p.Pro587Leu were additionally assessed using raw genotyping data, with carriers identified through PLINK v2.0. For individuals with both genotyping and WGS data available, carrier status was cross-validated across platforms to ensure concordance.

The pathogenicity and potential clinical significance of candidate variants were evaluated using multiple resources. Variants were included only if they met the following criteria: (i) located in coding or splice-affecting regions of POLG, including missense, predicted loss-of-function (stop-gain, stop-loss, frameshift, start-loss), or short insertion/deletion variants; (ii) Combined Annotation Dependent Depletion (CADD) score ≥12.37, corresponding to the top 2% of predicted pathogenic variants in the human genome;^29^ and (iii) predicted to be pathogenic or likely pathogenic by at least two of three tools: ClinVar,^30^ Franklin,^31^ and Varsome,^32^ the latter two based on the consensus guideline of the American College of Medical Genetics and Genomics (ACMG).^33^

### Burden analyses of rare *POLG* variants

To evaluate the cumulative contribution of rare *POLG* variants to PD risk, we performed gene-based burden analyses leveraging the WGS data (n=14,704 PD cases and 5,951 controls) using the sequence kernel association test (SKAT) and optimal unified SKAT (SKAT-O) methods as implemented in RVTESTS.^34^

Variants were grouped into ten categories for burden testing: (i) and (ii) all rare variants with MAF <1% and <0.1% according to gnomAD, respectively; (iii) and (iv) rare missense variants with MAF <1% and 0.1%, respectively; (v) and (vi) rare predicted loss-of-function variants (i.e., splicing, stop-gain, stop-loss, and frameshift) with MAF <1% and 0.1%, respectively; (vii) and (viii) rare damaging missense variants with MAF <1% and 0.1%, respectively, defined as CADD ≥20 and at least one of the following: REVEL score ≥0.644, Polyphen-2 predicted damaging, SIFT predicted damaging, or PrimateAI >0.8; (ix) and (x) rare variants classified as pathogenic or likely pathogenic in ClinVar with MAF <1% and 0.1%, respectively. Burden analyses were not performed for AAC, CAH, CAS, FIN, and SAS populations because of limited sample sizes among PD cases and controls.

### Case-control association of common *POLG* variants

We further investigated the association between common POLG variants and PD risk by analyzing NBA imputed genotyping data. We used PLINK v2.0 for variant extraction and ANNOVAR^28^ for variant annotation; only variants with an imputation quality score (R^2^) ≥0.8 were kept for further analyses.

Variants deviating from HWE in control samples (P-value ≤1 × 10^−5^) were excluded during preliminary QC. Additional pruning was applied to remove rare variants with MAF <1% based on gnomAD v4.1.1 (https://gnomad.broadinstitute.org/), as well as variants with a minor allele count (MAC) <2. SNP-phenotype association analyses were conducted using a generalized linear model implemented in PLINK v2.0.^35^ Logistic regression models included sex, age, and the first five genetic principal components (PCs) as covariates to account for population stratification. Bonferroni correction was applied to adjust p-values for all *POLG* variants within each ancestry group independently.

## Results

### Potentially biallelic pathogenic or likely pathogenic *POLG* variant carriers

Overall, five individuals with PD were identified harboring potentially biallelic pathogenic or likely pathogenic *POLG* variants (Table 1), although the phase of these variants could not be established to confirm compound heterozygosity.

One PD case of EUR ancestry (GP2-ID-1) was found to carry a homozygous pathogenic *POLG* variant, p.Gly737Arg. Another EUR PD case (GP2-ID-2) carried the pathogenic co-inherited *POLG* p.Thr251Ile and p.Pro587Leu variants in the heterozygous state, along with another heterozygous pathogenic *POLG* variant, p.Leu304Arg. The third EUR PD case (GP2-ID-3) carried p.Gly737Arg and p.Ala143Val variants, whereas the fourth EUR PD case (GP2-ID-4) harboured p.Pro648Arg and p.Ala467Thr variants. None of these carriers were related to each other, and no additional pathogenic variants in other genes linked to genetic PD were identified. Lastly, a PD case of AMR ancestry (GP2-ID-5) was homozygous for the co-inherited p.Thr251Ile and p.Pro587Leu variants, but exome sequencing also revealed a homozygous pathogenic *PRKN* c.1286–3C>G variant in this individual.

Clinically, there was a wide variability in the ages at onset (AAO) of the PD cases carrying potentially biallelic *POLG* pathogenic or likely pathogenic variants, ranging from 20 to 60 years. The case with the earliest AAO between 15–25 years (GP2-ID-5) also harbored a homozygous pathogenic *PRKN* variant (chr6:161350214:G:C [hg38]; c.1286–3C>G), a variant known to cause early-onset PD, which likely explains the early disease onset in this individual. Among all five carriers, two reported a negative family history of PD, two reported positive history, and one was unknown. Based on the available clinical data, all five patients had a diagnosis of typical PD without mention of relevant atypical features indicating a different diagnosis. Data on medication was only available for two individuals, both of whom showed a good response to dopaminergic treatment. In the context of non-motor features, one individual was found to have mild cognitive impairment (MCI; MoCA score 23/30). Furthermore, two PD cases were found to have comorbidities, including kidney disease and hypertension, respectively. A summary of clinical characteristics is provided in Table 1; overall, the availability of extended clinical data was limited.

### Single heterozygous pathogenic or likely pathogenic *POLG* variant carriers

**Figure 2:**
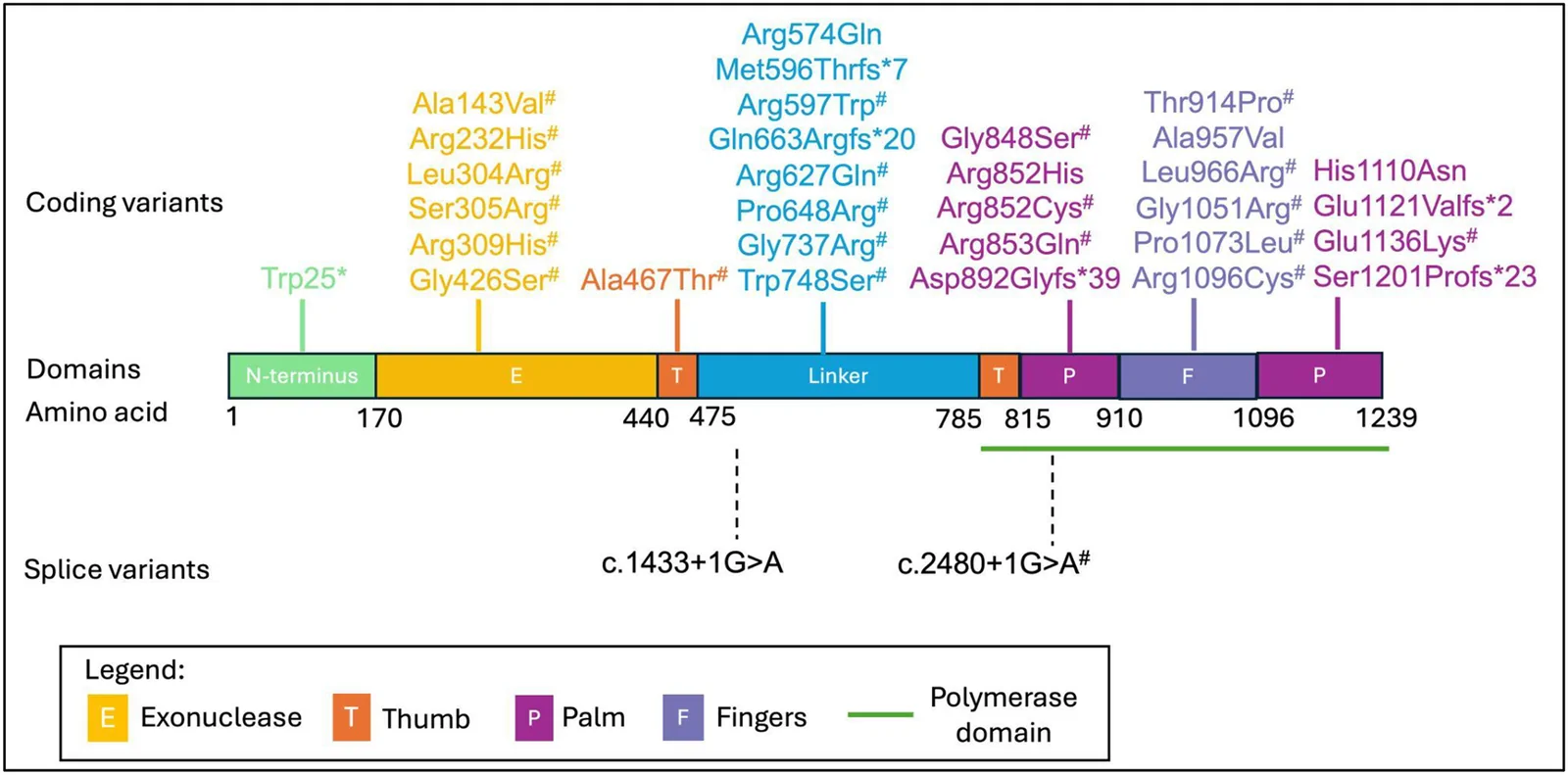
Pathogenic or likely pathogenic *POLG* variants identified across ancestries. Variants predicted to be pathogenic or likely pathogenic were illustrated and mapped onto the domains of POLG protein. The approximate positions of intronic variants, referenced to the protein domains, are indicated by dashed lines. ^#^Previously reported as disease-associated variants under both dominant and recessive inheritance (https://tools.niehs.nih.gov/polg/index.cfm/main/home).

We identified a total of 34 distinct pathogenic or likely pathogenic variants across ancestries in the heterozygous state, most of which were located within the polymerase domain of the POLG protein (Figure 2). A summary of all the variants and their respective frequencies is provided in Table 2; several variants were exclusively identified in PD cases and absent from controls. All variants have been reported before as disease-related for different neurological phenotypes, and the majority of them (n=24) have previously been linked to diseases with either dominant or recessive inheritance, according to the National Institute of Environmental Health Sciences (NIEHS) database (https://portal.niehs.nih.gov). Two of these variants were found in multiple individuals from two EUR families. The p.Gly737Arg variant was identified in two siblings, both affected with PD (AAO ranging 60–70 years, respectively), whereas p.Ala467Thr was found in a PD proband with a very early disease onset (AAO ranging 15–25 years) and his unaffected mother (aged ranging 60–70 years). Notably, both the individual with PD and his unaffected mother also carried a heterozygous pathogenic *PRKN* variant (p.Cys253Tyr). Overall, heterozygous pathogenic or likely pathogenic *POLG* variants were present in <1% of PD cases (n=161/23,315; 0.69%), affected individuals with other neurological disorders (n=28/3,974; 0.70%), and healthy controls (n=39/7,585; 0.51%) across all ancestries. Except for p.Ala467Thr, which was significantly enriched in PD cases in the EUR population (P=0.0443), no other variants showed a significant enrichment in PD.

### Carrier frequency of two co-inherited *POLG* variants in same haplotype

The *POLG* variants p.Thr251Ile and p.Pro587Leu were reported to co-occur on the same haplotype based on gnomAD, and are classified as pathogenic in combination according to ClinVar (Supplementary Figure 1). The carrier frequency of these co-inherited *POLG* variants was subsequently explored in PD cases, individuals with other neurological disorders, and controls across ancestries. Overall, the carrier frequency was <1% across ancestries and showed no significant differences between PD cases and controls (Table 3).

### Gene-based burden analysis

The results of the burden analysis across the investigated variant groups and ancestries are summarized in Supplementary Table 2. Nominal significance was observed between rare missense variants with a MAF <0.1% as well as damaging missense variants and PD in the EAS population (SKAT P=0.0208, SKAT-O P=0.0520 and SKAT-O P=0.0294, respectively). However, after multiple testing corrections, none of the rare *POLG* variants showed a significant burden association with PD.

### Case-control analysis of *POLG* variants

Of the 17 common (MAF ≥1%) exonic POLG variants retained after filtering, only one variant showed nominally significant allele frequency differences between PD cases and controls: p.Leu752= (chr15–89323415 G>A; rs41564016) in the EUR population (P=0.0418). However, the association was not significant after Bonferroni correction. A summary of all exonic POLG variants is provided in Supplementary Table 3.

## Discussion

In this study, we characterized the frequency and spectrum of *POLG* variants in PD and atypical parkinsonism compared to healthy controls across diverse ancestries. To our knowledge, this represents the largest multi-ancestry analysis to date examining the relationship between *POLG* genetic variation and PD. Upon screening short-read sequencing data from 36,022 individuals, we found that carriers of potentially biallelic pathogenic or likely pathogenic *POLG* variants were rare among PD cases (n=5) worldwide. Similarly, single heterozygous pathogenic or likely pathogenic variants occurred in <1% of both cases and controls across ancestries.

As a first step, we investigated potentially biallelic disease-causing *POLG* variants, and identified five PD cases carrying homozygous or multiple heterozygous pathogenic or likely pathogenic variants. Among these, the p.Gly737Arg variant was observed in two PD patients, either in the homozygous state (GP2-ID-1) or as a double heterozygote with the co-inherited p.Thr251Ile and p.Pro587Leu variants (GP2-ID-3). The p.Gly737Arg substitution lies within the linker region of the polymerase γ and has been implicated in a spectrum of mitochondrial disorders and neuropathies, including ataxia, seizures, and myopathy.^2^ It has been reported in compound heterozygosity with other *POLG* variants (e.g., p.Arg309His, p.Ser64Leu), and associated with axonal Charcot-Marie-Tooth-like neuropathy, occasional dystonia, and parkinsonism features.^36–38^ Notably, two sisters with early-onset PD and sensory neuropathy were previously reported to carry the p.Gly737Arg and p.Arg853Trp variants on different haplotypes (*trans*).^8^ Collectively, these findings underscore the broad clinical spectrum associated with the *POLG* p.Gly737Arg variant.

The co-inherited *POLG* p.Thr251Ile and p.Pro587Leu variants were identified either as double heterozygotes with p.Leu304Arg (GP2-ID-2), or in the homozygous state (GP2-ID-5). These two variants occur in complete linkage disequilibrium, forming a *cis* haplotype that has been described in multiple mitochondrial disease cohorts,^39–43^ either in homozygous cases,^43^ or, more commonly, in combination with other *POLG* variants.^44,45^ Functional studies show that each variant individually modestly impairs polymerase γ activity. However, when present together in *cis*, they act synergistically, reducing DNA-binding affinity and polymerase activity to approximately 5–10% of wild-type levels, leading to defective mtDNA replication and repair.^46^ Clinically, carriers of this haplotype typically present with PEO and multisystemic mitochondrial features, including ataxia, myopathy, epilepsy, neuropathy, and hepatic dysfunction.^41,46^

Clinically, all five PD cases with potentially biallelic *POLG* variants identified in our cohort were all diagnosed with typical PD, without obvious atypical features and with a good response to dopaminergic treatment in two individuals with available medication data. The ages at onset varied substantially, ranging from 20 to 60 years. Notably, one individual also carried a homozygous pathogenic *PRKN* variant, which is more likely to represent the disease-causing alteration in this case and is consistent with the very early onset ranging 15–25 years observed in this individual. While additional clinical data was very limited, particularly on non-motor features, one individual showed mild cognitive impairment. Interestingly, cognitive dysfunction has been previously reported in *POLG*-related disorders, including *POLG*-associated parkinsonism,^41,45^ although its frequency and severity appear to vary depending on the disease phenotype, age at onset, and underlying variant combination. Unfortunately, additional clinical information on variant carriers, including potential mitochondrial and systemic features, were unavailable, precluding us from investigating potential genotype-phenotype correlations in more detail. Based on the limited clinical data, the small number of affected individuals, and the uncertainty regarding true biallelic status due to the constraints of the available sequencing data, we cannot conclusively determine whether the identified *POLG* variants are causal, contributory, or merely coincidental in relation to the PD phenotype.

Investigating single heterozygous variants, we found the pathogenic p.Ala467Thr variant to be enriched in the EUR PD population compared to controls. This variant has been reported to occur in ~0.6% of the Belgian population, and is one of the most frequently detected *POLG* variants associated with Alpers syndrome, autosomal recessive PEO and SANDO.^47,48^ The variant is located within the linker region and has been found to reduce the catalytic efficiency of POLG via *in vitro* study.^49^ In addition, we have identified two variants (Gly737Arg and p.Ala467Thr) in multiple family members. *POLG* p.Gly737Arg was identified in two affected siblings with PD without any other known PD causative genetic variant, supporting a potential pathogenic role of this variant even in the heterozygous state. However, no additional family members were available to perform extended segregation analyses. In comparison, p.Ala467Thr was identified in both the affected PD proband and his unaffected mother, and both also carried a heterozygous pathogenic *PRKN* variant. Considering the early onset of the PD proband, this individual might harbor a second undetected pathogenic *PRKN* variant, which more likely represents the driver of the disease.

Overall, the role of heterozygous *POLG* variants in PD remains uncertain. While *POLG* is classically more commonly linked to recessive mitochondrial diseases, single pathogenic variants may act as reduced-penetrance risk alleles or require an additional genetic, environmental, or age-related “second hit”. This has important implications for clinical interpretation and particularly genetic counseling, as the presence of a single pathogenic *POLG* variant alone does not necessarily establish causality for PD. Future studies should therefore incorporate the systematic assessment of mitochondrial features to better estimate pathogenicity and distinguish incidental carrier status from *POLG*-related disease presenting with parkinsonism. Detailed phenotyping will be particularly important for counseling carriers, where current evidence often does not allow a clear distinction between causal, contributory, and incidental findings.

For the two co-inherited variants p.Thr251Ile and p.Pro587Leu, carriers were identified in <1% of both PD cases and controls, with no significant difference between groups. The overall frequency observed in controls aligns with a previous report from an Italian control population (~1%); however, the prevalence in PD was not assessed in that study.^42^ To our knowledge, our study is the first to examine the frequency of these two co-inherited variants across ancestries and at a large scale. Despite their predicted pathogenicity, the absence of significant enrichment among PD cases suggests that this haplotype is unlikely to represent a major genetic factor relevant to PD.

In our single variant case-control association analyses, no common *POLG* variants showed a significant association with PD after correction for multiple testing, consistent with prior reports that found no robust evidence linking *POLG* variation to PD risk.^15,16,19^ These results further indicate that common *POLG* variants likely play a limited role in PD susceptibility.

Finally, our burden analyses using WGS data revealed significant association between rare *POLG* variant burden and PD in the AAC and SAS populations. While some trends can also be observed in other ancestral groups, these findings should be interpreted with caution due to the small sample sizes across most ancestral groups, which may reduce statistical power and affect the robustness of these estimates. Further studies incorporating larger sequencing cohorts will be essential to comprehensively characterize the contribution of rare *POLG* variants to PD pathogenesis.

There are several limitations in this study. First, certain ancestry groups, particularly AAC, FIN and SAS, had relatively small sample sizes compared to other populations, reducing statistical power to detect rare variant associations. Second, phasing information for potential biallelic variants (*cis* or *trans* configuration) was unavailable, limiting assessment of compound heterozygosity. Third, the lack of detailed clinical data for carriers of potentially pathogenic or likely pathogenic *POLG* variants prevented in-depth genotype-phenotype correlation, particularly regarding potential mitochondrial features. Finally, the study did not assess potential CAG repeat expansions within *POLG*, and individuals with classical mitochondrial *POLG*-related phenotypes (e.g., myopathy, PEO, or SANDO) accompanied by parkinsonism may be underrepresented in this dataset due to GP2 recruitment criteria primarily targeting PD cases.

In conclusion, pathogenic *POLG* variants are rare among individuals presenting with typical PD and show no overall enrichment compared to controls across ancestries. The identification of cases carrying potentially biallelic pathogenic variants suggests that *POLG*-related mitochondrial dysfunction may contribute to disease in isolated instances, supporting a possible role for recessive *POLG* alterations in rare forms of PD while the contribution of heterozygous variants to PD risk still remains partially elusive and requires further investigation using even larger, high-quality sequencing datasets. While heterozygous *POLG* variants did not show variant-level associations with PD, the identification of rare pathogenic alleles among cases and the nominal burden signals for rare variants point to a potential, albeit limited, contribution of rare *POLG* variation to PD susceptibility and underscore the continued relevance of mitochondrial dysfunction in PD. Given the central role of *POLG* in mtDNA replication and repair, and the occurrence of parkinsonian features in some *POLG*-related disorders, further investigation is warranted to clarify genotype-phenotype relationships. Future studies involving detailed phenotypic characterization of PD cases with *POLG* variants, and functional assays assessing variant effects on mitochondrial and neuronal function will be essential to delineate the contribution of *POLG* to PD biology.

## Supplementary Material

Supplement 1

## Figures and Tables

**Figure 1:**
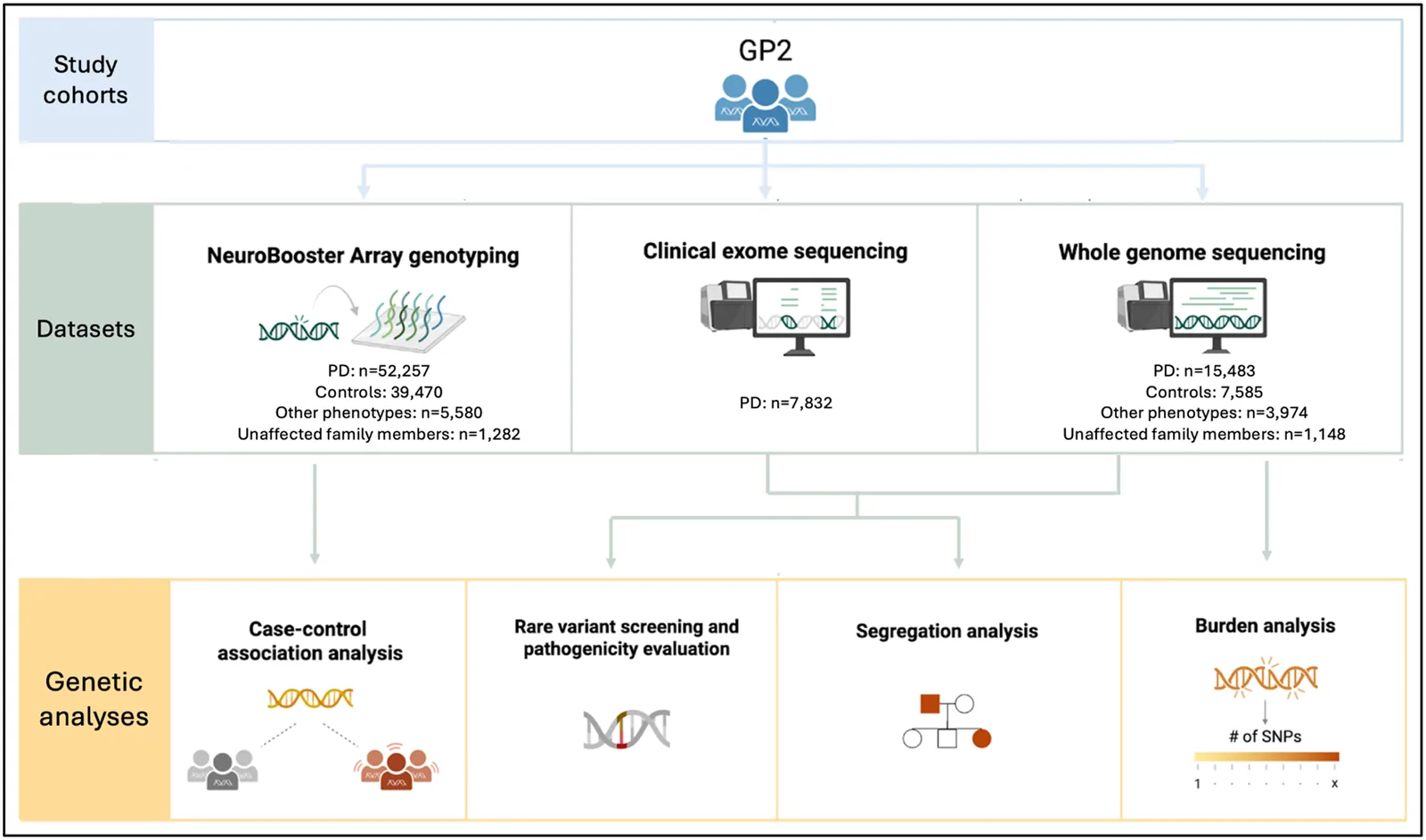
Overview of the study workflow. Data from GP2 release 11 (NBA, CES, and WGS) were leveraged and underwent quality control. Variants in the *POLG* gene were extracted and annotated. Downstream analyses included a case-control association analysis, gene-based burden analysis of rare *POLG* variants, screening for rare *POLG* variants (biallelic and monoallelic), and segregation analysis. CES = Clinical exome sequencing; NBA = NeuroBooster Array genotyping; WGS = Whole genome sequencing.

**Table 1:** Carriers of potentially biallelic pathogenic or likely pathogenic *POLG* variants identified across ancestries.

	GP2-ID-1	GP2-ID-2	GP2-ID-3	GP2-ID-4	GP2-ID-5
Genetic Details					
*POLG variants*	chr15:89323460:C:G (p.Gly737Arg)^#^	1) chr15:89329055:A:C (p.Leu304Arg)^#^ 2) chr15:89330184:G:A (p.Thr251Ile)^#^ 3) chr15:89325639:G:A (p.Pro587Leu)^#^	1) chr15:89323460:C:G (p.Gly737Arg)^#^ 2)chr15:89333327:G:A (p.Ala143Val)^#^	1) chr15:89325456:G:C (p.Pro648Arg)^#^ 2)chr15:89327201:C:T (p.Ala467Thr)^#^	1) chr15:89330184:G:A (p.Thr251Ile)^#^ 2) chr15:89325639:G:A (p.Pro587Leu)^#^
Zygosity	Hom	Potential compound het	Potential compound het	Potential compound het	Double hom
Pathogenicity (ClinVar)	Pathogenic/Likely Pathogenic	1) Pathogenic/Likely Pathogenic 2) + 3) Pathogenic	1) + 2) Pathogenic/Likely Pathogenic	1) + 2) Pathogenic/Likely Pathogenic	1) + 2) Pathogenic
Genetic screening method	CES	CES	CES	WGS	CES
Other genetic findings	NA	NA	NA	NA	*PRKN*: c.1286–3C>G (Hom); pathogenic
Basic Demographics					
Genetic ancestry	EUR	EUR	EUR	EUR	AMR
Gender	Male	Male	Female	Male	Male
Age at sample collection, range in years	60–70	40–50	65–75	45–55	40–50
Clinical Characteristics					
Age at onset, range in years	50–60	35–45	55–65	40–50	15–25
Age at most-recent follow-up, range years	60–70	40–50	NA	NA	40–50
Family history of PD	No	No	Unknown	Yes	Yes
Diagnosis	PD	PD	PD	PD	PD
Levodopa responsiveness	Unknown	Yes	Unknown	Unknown	Yes
Cognition	Unknown	MCI MoCA 23/30 points	Unknown	Unknown	Self-reported MCI MoCA 28/30 points
Other comorbidities	Unknown	Kidney disease	Unknown	Unknown	Hypertension

# Previously reported as disease-associated variants under both dominant and recessive inheritance (https://tools.niehs.nih.gov/polg/index.cfm/main/home).

AMR = Latinos and Indigenous People of the Americas, CES = Clinical exome sequencing, EUR = European, Het = Heterozygous, Hom = Homozygous, MCI = Mild cognitive impairment, MoCA = Montreal cognitive assessment, NA = Not available/applicable.

**Table 2:** Carriers of single heterozygous pathogenic or likely pathogenic *POLG* variants across ancestry groups.

Variant (hg38)	Pathogenicity (ClinVar)	Ancestry	PD (Freq,%)	Other (Freq, %)	Control (Freq, %)	P value^a^	MAF gnomAD^b^
chr15:89333680:C:T (p.Trp25*)	P	EUR	0/8044 (0)	0/3264 (0)	1/2108 (0.05)	NA	8.68E-07
chr15:89333327:G:A (p.Ala143Val)^#^	P/LP	CAS	1/663 (0.15)	0/165 (0)	0/957 (0)	NA	NA
		EUR	4/8044 (0.05)	0/3264 (0)	1/2108 (0.05)	1	0.00004338
chr15:89330241:C:T (p.Arg232His)^#^	P	EAS	0/2697 (0)	0/211 (0)	1/1115 (0.09)	NA	0
chr15:89329055:A:C (p.Leu304Arg)^#^	P/LP	CAH	1/177 (0.56)	0/50 (0)	0/128 (0)	NA	NA
		CAS	1/663 (0.15)	0/165 (0)	0/957 (0)	NA	NA
		EUR	2/8044 (0.02)	0/3264 (0)	1/2108 (0.05)	0.5026	0.000005085
chr15:89329051:G:C (p.Ser305Arg)^#^	P/LP	EUR	0/8044 (0)	0/3264 (0)	1/2108 (0.05)	NA	0.000007627
chr15:89329040:C:T (p.Arg309His)^#^	P	EUR	1/8044 (0.01)	0/3264 (0)	0/2108 (0)	NA	0.00001017
chr15:89327324:C:T (p.Gly426Ser)^#^	P/LP	AJ	1/741 (0.13)	0/114 (0)	0/669 (0)	NA	0.0001351
chr15:89327201:C:T (p.Ala467Thr)^#^	P	AMR	1/338 (0.30)	0/20 (0)	0/182 (0)	NA	0.00004998
		EUR	42/8044 (0.52)	2/3264 (0.06)	4/2108 (0.19)	**0.0443**	0.0001732
chr15:89325678:C:T (p.Arg574Gln)	P/LP	CAS	0/663 (0)	0/165 (0)	1/957 (0.10)	NA	NA
		EUR	1/8044 (0.01)	0/3264 (0)	0/2108 (0)	NA	0.00004665
		SAS	0/447 (0)	0/67 (0)	2/360 (0.56)	NA	0.00002196
chr15:89325612:A:ATCTG (p.Met596Thrfs*7)	P	EUR	1/8044 (0.01)	0/3264 (0)	0/2108 (0)	NA	NA
chr15:89325610:G:A (p.Arg597Trp)^#^	P/LP	AFR	1/1469 (0.07)	0/38 (0)	1/2097 (0.05)	1	0.00007996
		EAS	0/2697 (0)	0/211 (0)	1/1115 (0.09)	NA	0
		EUR	1/8044 (0.01)	0/3264 (0)	0/2108 (0)	NA	0.000002542
chr15:89324187:CCT:C (p.Gln663Argfs*20)	P/LP	AJ	0/741 (0)	0/114 (0)	1/669 (0.15)	NA	0
		EUR	1/8044 (0.01)	0/3264 (0)	0/2108 (0)	NA	0.000001695
chr15:89325519:C:T (p.Arg627Gln)^#^	P/LP	EUR	0/8044 (0)	1/3264 (0.03)	2/2108 (0.09)	NA	0.00003051
chr15:89325456:G:C (p.Pro648Arg)^#^	P/LP	EUR	1/8044 (0.01)	0/3264 (0)	0/2108 (0)	NA	0.00001104
chr15:89323460:C:G (p.Gly737Arg)^#^	P/LP	AAC	0/190 (0)	1/13 (7.69)	0/129 (0)	NA	0.0004263
		AMR	1/338 (0.30)	0/20 (0)	0/182 (0)	NA	0.0002833
		EUR	45/8044 (0.56)	13/3264 (0.40)	10/2108 (0.47)	0.7403	0.002029
chr15:89323426:C:G (p.Trp748Ser)^#^	P/LP	AMR	1/338 (0.30)	0/20 (0)	0/182 (0)	NA	0.0001333
		CAS	0/663 (0)	1/165 (0.61)	0/957 (0)	NA	0.006326
		EUR	12/8044 (0.15)	2/3264 (0.06)	2/2108 (0.09)	0.7479	0.0006115
		SAS	1/447 (0.22)	0/67 (0)	0/360 (0)	NA	0.00005491
chr15:89321792:C:T (p.Gly848Ser)^#^	P	AFR	0/1469 (0)	0/38 (0)	3/2097 (0.14)	NA	0.0001866
		EUR	13/8044 (0.16)	6/3264 (0.18)	2/2108 (0.09)	0.7504	0.0003941
chr15:89321779:C:T (p.Arg852His)	LP	EAS	1/2697 ( 0.04)	0/211 (0)	0/1115 (0)	NA	0
chr15:89321780:G:A (p.Arg852Cys)^#^	P	EUR	5/8044 (0.06)	1/3264 (0.03)	2/2108 (0.09)	0.6402	0.0001424
chr15:89321776:C:T (p.Arg853Gln)^#^	P/LP	EUR	1/8044 (0.01)	0/3264 (0)	0/2108 (0)	NA	0.000002542
chr15:89321184:T:TC (p.Asp892Glyfs*39)	P	EUR	1/8044 (0.01)	0/3264 (0)	0/2108 (0)	NA	0.00000339
chr15:89321007:T:G (p.Thr914Pro)^#^	P/LP	EUR	3/8044 (0.04)	0/3264 (0)	0/2108 (0)	NA	0.00025
chr15:89320877:G:A (p.Ala957Val)	P/LP	EUR	1/8044 (0.01)	0/3264 (0)	0/2108 (0)	NA	8.47E-07
chr15:89320850:A:C (p.Leu966Arg)^#^	P/LP	EUR	1/8044 (0.01)	1/3264 (0.03)	0/2108 (0)	NA	0.00005678
chr15:89319053:C:G (p.Gly1051Arg)^#^	P/LP	EUR	2/8044 (0.02)	0/3264 (0)	1/2108 ( 0.05)	0.5026	0.00004153
		MDE	2/694 (0.29)	0/26 (0)	0/984 (0)	NA	0.0004949
chr15:89319053:C:T (p.Gly1051Arg)^#^	P/LP	EAS	1/2697 (0.04)	0/211 (0)	0/1115 (0)	NA	0.00001356
chr15:89318986:G:A (p.Pro1073Leu)^#^	P	AFR	1/1469 (0.07)	0/38 (0)	0/2097 (0)	NA	0
		EAS	2/2697 (0.07)	0/211 (0)	0/1115 (0)	NA	0.00002228
chr15:89318737:G:A (p.Arg1096Cys)^#^	LP	CAS	0/663 (0)	0/165 (0)	1/957 (0.10)	NA	NA
		MDE	1/694 (0.14)	0/26 (0)	0/984 (0)	NA	0
chr15:89318695:G:T (p.His1110Asn)	VUS/LP	EUR	1/8044 (0.01)	0/3264 (0)	0/2108 (0)	NA	NA
chr15:89318661 :T:TCAAA (p.Glu1121Valfs*2)	P/LP	EUR	1/8044 (0.01)	0/3264 (0)	0/2108 (0)	NA	8.47E-07
chr15:89318617:C:T (p.Glu1136Lys)^#^	P/LP	EUR	1/8044 (0.01)	0/3264 (0)	0/2108 (0)	NA	0.000002542
chr15:89317417:GA:G (p.Ser1201Profs*23)	P/LP	EUR	1/8044 (0.01)	0/3264 (0)	0/2108 (0)	NA	0.00000339
chr15:89327166:C:T (c.1433+1G>A)	P	AFR	1/1469 (0.07)	0/38 (0)	1/2097 (0.05)	1	0.0001468
chr15:89321961:C:T (c.2480+1G>A)^#^	P	EAS	1/2697 (0.04)	0/211 (0)	0/1115 (0)	NA	0

a Fisher’s exact test was used to compare the frequency of the *POLG* variants among PD cases and controls.

b For each variant, we reported the ancestry-specific minor allele frequency (MAF) based on data from gnomAD v4.1.0. Frequencies of ancestry groups in our study without corresponding groups available on gnomAD are labeled as NA.

# Previously reported as disease-associated variants under both dominant and recessive inheritance (https://tools.niehs.nih.gov/polg/index.cfm/main/home).

AAC = African Admixed, AFR = African, AJ = Ashkenazi Jewish, AMR = Latinos and Indigenous People of the Americas, CAH = Complex Admixture History, CAS = Central Asian, EAS = East Asian, EUR = European, Freq = Frequency, FIN = Finnish, MAF = minor allele frequency, LP = Likely pathogenic, MDE = Middle Eastern, NA = Not available/applicable, P = Pathogenic, SAS = South Asian, VUS = Variant of uncertain significance, WGS = Whole genome sequencing.

**Table 3:** Carrier frequency of co-inherited *POLG* p.Thr251Ile and p.Pro587Leu variants across ancestries.

Ancestry	NBA				WGS			
	PD (Freq,%)	Other (Freq,%)	Control (Freq,%)	P value^a^	PD (Freq,%)	Other (Freq,%)	Control (Freq,%)	P value^a^
AAC	1/491 (0.20)	0/18 (0.00)	1/846 (0.12)	1.000	0/190 (0.00)	0/13 (0.00)	0/129 (0.00)	NA
AFR	0/2770 (0.00)	0/44 (0.00)	1/4412 (0.02)	NA	0/1469 (0.00)	0/38 (0.00)	0/2097 (0.00)	NA
AJ	3/2225 (0.13)	0/29 (0.00)	0/801 (0.00)	NA	2/741 (0.27)	0/114 (0.00)	4/669 (0.60)	0.4314
AMR	7/2222 (0.32)	0/28 (0.00)	0/1508 (0.00)	NA	0/338 (0.00)	0/20 (0.00)	0/182 (0.00)	NA
CAH	NA	NA	NA	NA	1/177 (0.56)	0/50 (0.00)	0/128 (0.00)	NA
CAS	2/1194 (0.17)	0/175 (0.00)	1/1373 (0.07)	0.6006	1/663 (0.15)	0/165 (0.00)	0/957 (0.00)	NA
EAS	0/4609 (0.00)	0/396 (0.00)	0/2769 (0.00)	NA	0/2697 (0.00)	0/211 (0.00)	0/1115 (0.00)	NA
EUR	129/35955 (0.36)	15/4625 (0.32)	96/25598 (0.38)	0.7354	67/8044 (0.83)	19/3264 (0.58)	14/2108 (0.66)	0.4940
FIN	0/113 (0.00)	0/11 (0.00)	0/19 (0.00)	NA	0/23 (0.00)	0/6 (0.00)	0/4 (0.00)	NA
MDE	2/1100 (0.18)	1/35 (2.86)	2/1304 (0.15)	1.000	2/694 (0.29)	0/26 (0.00)	4/984 (0.41)	1.000
SAS	0/739 (0.00)	0/103 (0.00)	0/398 (0.00)	NA	0/447 (0.00)	0/67 (0.00)	0/360 (0.00)	NA

a Fisher’s exact test was used to compare the frequency of the two *POLG* variants among PD cases and controls. Comparison was not available when no event was observed in either group.

AAC = African Admixed, AFR = African, AJ = Ashkenazi Jewish, AMR = Latinos and Indigenous People of the Americas, CAH = Complex Admixture History, CAS = Central Asian, EAS = East Asian, EUR = European, Freq = Frequency, FIN = Finnish, NBA = NeuroBooster Array, MDE = Middle Eastern, NA = Not available/applicable, SAS = South Asian, WGS = Whole genome sequencing.

## Data Availability

Data used in the preparation of this article were obtained from the Global Parkinson’s Genetics Program (GP2; https://gp2.org). Specifically, we used Tier 2 data from GP2 release 11 (10.5281/zenodo.17753486). GP2 data can be accessed through AMP PD (https://amp-pd.org).

## Code Availability

All code generated for this article, and the identifiers for all software programs and packages used, are available on GitHub (https://github.com/GP2code/POLG_multiancestry) and were given a persistent identifier via Zenodo (10.5281/zenodo.19861264).
