# Supplementary material for: Multi-ancestry analysis of POLG variants in Parkinson’s disease
Source: medRxiv. 2026 Jun 8:2026.06.07.26354811. Preprint. [Version 1] doi: 10.64898/2026.06.07.26354811 (PMC13278229; doi:10.64898/2026.06.07.26354811)
Supplement: Supplementary file 1 [file media-1.pdf]

Supplementary Material

Multi-ancestry analysis of *POLG* variants in Parkinson's disease

Yi Wen Tay, PhD [1a]; Inas Elsayed, PhD [2,3a]; Dennis Yeow, BSc, MBBS, FRACP [4,5,6,7]; Mikayla James, MSc [8]; Pin-Jui Kung, PhD [9]; Laurel Screven, PhD [10]; Allison A. Dilliott, PhD [11]; Roy N. Alcalay, MD, MS [12]; Zih-Hua Fang, PhD [13]; Ai Huey Tan, MD, PhD, FRCP [14]; the Global Parkinson's Genetics Program (GP2); Carolyn M Sue, MBBS, PhD, FRACP [4,15,16,#]; Lara M Lange, MD [17,18,#]; Maria Teresa Periñan, PhD [19,20,#]

[1]Department of Biomedical Science, Faculty of Medicine, University of Malaya, Kuala Lumpur, Malaysia
[2]Faculty of Pharmacy, University of Gezira, Wad Medani, Sudan
[3]Systems Biology Ireland, School of Medicine and Medical Science, University College Dublin, Dublin, Ireland
[4]Neuroscience Research Australia, Randwick, New South Wales, Australia
[5]Translational Neurogenomics Group, Garvan Institute of Medical Research, Sydney, NSW, Australia
[6]Molecular Medicine Laboratory and Neurology Department, Concord Repatriation General Hospital, Concord, NSW, Australia
[7]Faculty of Medicine and Health, University of Sydney, Camperdown, Australia
[8]Center for Alzheimer's and Related Dementias (CARD), National Institute on Aging and National Institute of Neurological Disorders and Stroke, National Institutes of Health, Bethesda, MD, USA
[9]Division of Plastic Surgery, Department of Surgery, National Taiwan University Hospital, Taipei, Taiwan
[10]The Global Parkinson's Genetics Program (GP2)
[11]Parkinson's Foundation, NewYork, NY 10018, USA
[12]Neurological Institute, Tel Aviv Sourasky Medical Center, Tel Aviv, Israel and Department of Neurology, Columbia University Irving Medical Center, NYC, NY 10032, USA
[13]DataTecnica LLC, Washington, DC 20037, USA
[14]Division of Neurology, Department of Medicine, and The Mah Pooi Soo & Tan Chin Nam Centre for Parkinson's & Related Disorders, Faculty of Medicine, University of Malaya, Kuala Lumpur, Malaysia
[15]Faculty of Medicine, University of New South Wales, Sydney, NSW, Australia
[16]Department of Neurology, South Eastern Sydney Local Health District, Sydney, NSW, Australia
[17]Laboratory of Neurogenetics, National Institute on Aging, National Institutes of Health, Bethesda, MD, USA
[18]Institute of Neurogenetics, University of Luebeck, Luebeck, Germany
[19]Centre for Preventive Neurology, Wolfson Institute of Population Health, Queen Mary University of London, London, United Kingdom
[20]Unidad de Trastornos del Movimiento, Servicio de Neurología y Neurofisiología Clínica, Instituto de Biomedicina de Sevilla, Hospital Universitario Virgen del Rocío/Consejo Superior de Investigaciones Científicas (CSIC)/Universidad de Sevilla, Seville, Spain

[#]Equal contribution
[a]Joint first authors

Correspondence to:

Maria Teresa Periñan, PhD
Unidad de Trastornos del Movimiento, Servicio de Neurología y Neurofisiología Clínica, Instituto de Biomedicina de Sevilla, Hospital Universitario Virgen del Rocío/Consejo Superior de Investigaciones Científicas (CSIC)/Universidad de Sevilla, Seville, Spain
Centre for Preventive Neurology, Wolfson Institute of Population Health, Queen Mary University of London, London, United Kingdom
E-mail: t.perinantocino@qmul.ac.uk
and
Lara M. Lange, MD
Laboratory of Neurogenetics, National Institute on Aging,
9000 Rockville Pike, Bethesda, MD 20892, USA
Email: lara.lange@nih.gov

1. **Supplementary Figures ……………………………………………………………… 3**
2. **Supplementary Tables …………………………………………………………….. 5**

Supplementary Figures

A.

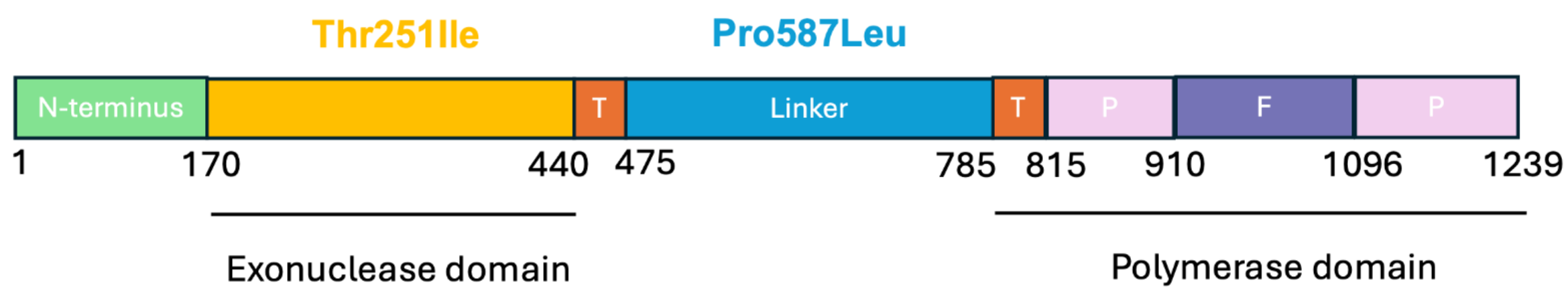

B. **chr15:89330184:G:A (p.Thr251Ile)** **chr15:89325639:G:A (p.Pro587Leu)**

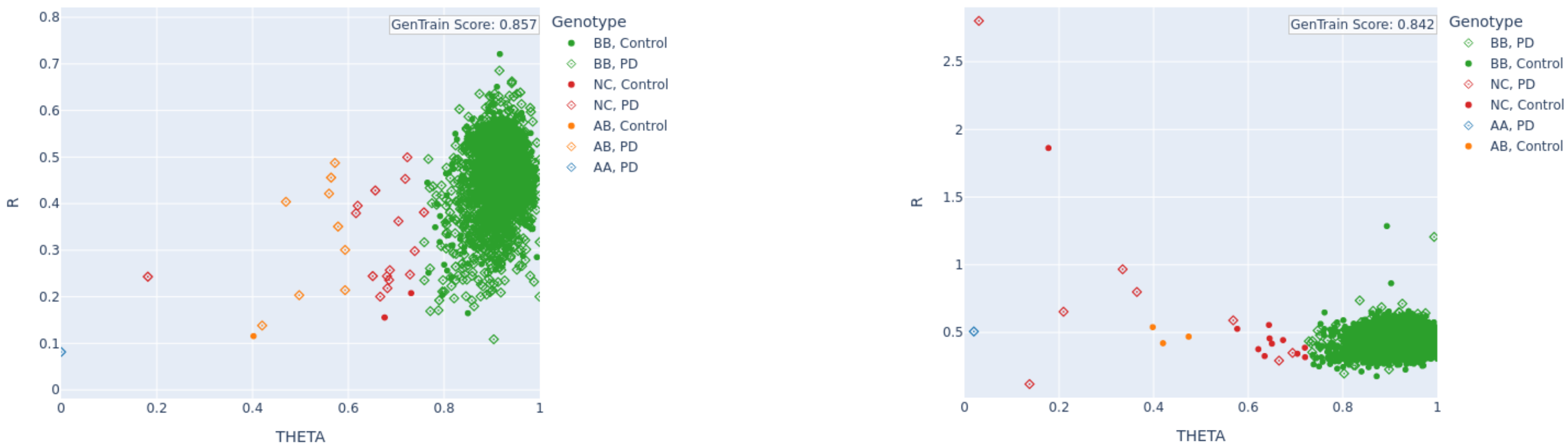

C.

rs113994094
chr15:89330184

rs113994096 chr15:89325639		A	G		
	A	4	0	4	(0.001)
	G	0	5002	5002	(0.999)
		4 (0.001)	5002 (0.999)	5006	

Haplotypes	Statistics
G_G: 5002 (0.999)	D': 1.0
A_A: 4 (0.001)	R^2: 1.0
G_A: 0 (0.0)	Chi-sq: 5006.0
A_G: 0 (0.0)	p-value: <0.0001

rs113994096(A) allele is correlated with rs113994094(A) allele
rs113994096(G) allele is correlated with rs113994094(G) allele

D.

Ancestry	Samples with variants in *trans*	Samples with variants in *cis*	Samples with variants in either *cis* or *trans*	Predicted pattern
AAC	0	0	4	Same haplotype
AMR	5	0	26	Same haplotype
AJ	0	0	20	Same haplotype
EAS	0	0	0	Same haplotype
FIN	0	0	7	Same haplotype
EUR	8	1	301	Same haplotype
Others	0	0	14	Same haplotype
SAS	6	0	3	Same haplotype
Total	19	1	375	Same haplotype

Supplementary Figure 1: Co-inherited *POLG* variants p.Thr251Ile and p.Pro587Leu. (A) The two co-inherited *POLG* variants [p.Thr251Ile (chr15:89330184:G:A; rs113994094) and p.Pro587Leu (chr15:89325639:G:A; rs113994096)] were mapped onto the domains of the POLG protein. (B) To ensure probe quality, cluster plots for the two variants genotyped on the NBA were visualized. (C) Co-occurrence information from the gnomAD database shows that the A alleles of both variants are inherited on the same haplotype. (D) Ancestry-stratified data adapted from gnomAD show a consistent co-occurrence across populations, supporting their co-inheritance. AAC = African Admixed, AJ = Ashkenazi Jewish, AMR = Latinos and Indigenous People of the Americas, EAS = East Asian, EUR = European, Freq = Frequency, FIN = Finnish, NBA = NeuroBooster Array, MDE = Middle Eastern, SAS = South Asian, WGS = Whole genome sequencing.

Supplementary Tables

Supplementary Table 1: Demographics of the studied cohorts from GP2 Release 11 data.

Genetic ancestry	Data type	Number of subjects, n			
		N PD [1]	*N* Other phenotypes [2]	*N* Control [3]	*N* Unaffected [4]
AAC	NBA	491	18	846	3
	WGS	190	13	127	2
	CES	147	0	0	0
AFR	NBA	2772	44	4413	1
	WGS	1469	38	2097	0
	CES	96	0	0	0
AJ	NBA	2238	111	804	525
	WGS	741	114	162	507
	CES	800	0	0	0
AMR	NBA	2229	28	1508	13
	WGS	338	20	170	12

	CES	320	0	0	0
CAH	NBA	804	33	409	8
	WGS	177	50	120	8
	CES	398	0	0	0
CAS	NBA	1195	175	1373	54
	WGS	663	165	913	44
	CES	38	0	0	0
EAS	NBA	4618	397	2791	70
	WGS	2697	211	1049	66
	CES	94	0	0	0
EUR	NBA	35955	4625	25598	552
	WGS	8044	3264	1651	457
	CES	5802	0	0	0
FIN	NBA	114	11	20	0

	WGS	23	6	4	0
	CES	16	0	0	0
MDE	NBA	1101	35	1309	11
	WGS	694	26	976	8
	CES	42	0	0	0
SAS	NBA	740	103	399	45
	WGS	447	67	316	44
	CES	79	0	0	0
**Total**	**NBA**	**52,257**	**5,580**	**39,470**	**1,282**
	**WGS**	**15,483**	**3,974**	**7,585**	**1,148**
	**CES**	**7,832**	**0**	**0**	**0**

[1] The "PD" category includes individuals with PD from i) unselected PD case/control cohorts, ii) monogenic recruitment, i.e., targeted recruitment of individuals in which a monogenic cause of disease is more likely based on a young age at onset <50 years and/or a positive family history of PD), and iii) genetically enriched cohorts, i.e., targeted recruitment of carriers of pathogenic variants in *GBA1*, *LRRK2*, or *SNCA*.

[2] The “Other phenotypes” category summarizes individuals with phenotypes other than classical PD, including atypical forms of parkinsonism (e.g., multisystem atrophy, progressive supranuclear palsy, corticobasal syndrome, and dementia with lewy bodies) and other neurodegenerative phenotypes (e.g., Alzheimer’s disease, mild cognitive impairment, other forms of dementia).

[3] The “control” category summarizes individuals without PD or other neurological/neurodegenerative phenotypes unrelated to individuals with PD or other phenotypes included in this study.

[4] The “unaffected” category includes unaffected family members of individuals with PD or other phenotypes included in this study.

AAC = African Admixed, AFR = African, AJ = Ashkenazi Jewish, AMR = Latinos and Indigenous People of the Americas, CAH = Complex Admixture History, CAS = Central Asian, CES = Clinical exome sequencing, EAS = East Asian, EUR = European, Freq = Frequency, FIN = Finnish, NBA = NeuroBooster Array, MDE = Middle Eastern, SAS = South Asian, WGS = Whole genome sequencing.

Supplementary Table 2: Burden analyses of *POLG* variants across ancestries.

Ancestry	N			MAF <1%														
				All			Missense			LOF			Damaging missense			P/LP		
	PD cases	Controls	Total	SKAT	SKATO	N variant	SKAT	SKATO	N variant	SKAT	SKATO	N variant	SKAT	SKATO	N variant	SKAT	SKATO	N variant
AFR	1,202	2,049	3,251	0.7746	0.8641	96	0.4246	0.3454	12	NA	NA	1	0.0905	0.1341	6	0.3795	0.1925	2
AJ	741	176	917	0.2219	0.3552	34	0.6676	0.8146	8	NA	NA	0	0.5336	0.4843	2	NA	NA	0
AMR	700	296	996	0.7837	1.000	41	0.9443	0.8309	10	NA	NA	1	0.8340	0.4411	4	NA	NA	0
EAS	1,347	981	2,328	0.2731	0.4416	81	0.1383	0.2496	17	NA	NA	0	0.2603	0.4209	9	NA	NA	0
EUR	10,087	2,040	12,127	0.8757	1.000	225	0.7331	0.8927	55	0.5525	0.7454	11	0.5556	0.7445	28	0.5525	0.7454	11
MDE	627	409	1,036	0.1164	0.1674	58	0.9852	1.000	11	NA	NA	0	0.9372	0.8410	5	NA	NA	1

Ancestry	N			MAF <0.1%														
				All			Missense			LOF			Damaging missense			P/LP		
	PD cases	Controls	Total	SKAT	SKATO	N variant	SKAT	SKATO	N variant	SKAT	SKATO	N variant	SKAT	SKATO	N variant	SKAT	SKATO	N variant
AFR	1,202	2,049	3251	0.4049	0.5875	61	0.5244	0.6528	8	NA	NA	1	0.4039	0.5137	5	0.3795	0.1925	2
AJ	741	176	917	0.5342	0.7074	14	NA	NA	1	NA	NA	0	NA	NA	0	NA	NA	0
AMR	700	296	996	0.7310	0.6106	11	NA	NA	1	NA	NA	1	NA	NA	0	NA	NA	0
EAS	1,347	981	2328	0.2043	0.2729	53	0.0208	0.0520	15	NA	NA	0	0.1287	0.0294	8	NA	NA	0
EUR	10,087	2,040	12,127	0.6242	0.6323	175	0.8658	1.000	43	0.3734	0.3988	9	0.4261	0.6404	22	0.3734	0.3988	9
MDE	627	409	1036	0.0991	0.0621	25	0.9083	0.8952	3	NA	NA	0	0.8347	0.7802	2	NA	NA	1

Gene-based burden analyses were performed using GP2 Release 11 whole genome sequencing data to evaluate the cumulative contribution of *POLG* variants to PD risk. The number of variants included in the analyses is reported for each ancestry group. P values that reached multiple correction thresholds were highlighted in bold.

AFR = African, AJ = Ashkenazi Jewish, AMR = Latinos and Indigenous People of the Americas, EAS = East Asian, EUR = European, Freq = Frequency, MAF = Minor allele frequency, MDE = Middle Eastern, N = Number, NA = Not available, SKAT = Sequence kernel association test, SKATO = Sequence kernel association test - optimal.

Supplementary Table 3: Association analysis of common *POLG* exonic variants using GP2 Release 11 genotyping data.

Variant	GP2 Ancestry	A1	A1 freq	OR (95% CI)	P-value (BONF)
chr15:89316763:C:A (p.Gln1236His)	AJ	A	0.0447	0.9271 (0.5880-1.4617)	0.7444 (1.000)
	AMR		0.0285	0.9956 (0.7002-1.4156)	0.9804 (1.000)
	CAS		0.0309	1.1307 (0.7108-1.7986)	0.6040 (1.000)
	EUR		0.0798	1.0331 (0.9722-1.0979)	0.2939 (1.000)
	MDE		0.0741	1.0986 (0.7896-1.5286)	0.5767 (1.000)
	SAS		0.0117	2.3475 (0.6639-8.3008)	0.1854 (1.000)
chr15:89317422:G:T (p.Thr1199=)	AJ	T	0.0137	0.5456 (0.2656-1.1209)	0.0991 (1.000)
	EUR		0.0106	1.0600 (0.9002-1.2481)	0.4847 (1.000)
chr15:89317458:C:G (p.Arg1187=)	AAC	G	0.0174	1.3915 (0.7005-2.7643)	0.3454 (1.000)
	AFR		0.0319	0.8986 (0.7259-1.1122)	0.3257 (1.000)
chr15:89318595:T:C (p.Glu1143Gly)	AAC	C	0.0115	1.0699 (0.4615-2.4805)	0.8748 (1.000)
	AJ		0.0543	0.8528 (0.5732-1.2686)	0.4319 (1.000)
	CAS		0.0260	0.9531 (0.5856-1.5512)	0.8466 (1.000)
	EUR		0.0414	0.9856 (0.9078-1.0699)	0.7284 (1.000)
	MDE		0.0325	0.8082 (0.4973-1.3134)	0.3900 (1.000)
	SAS		0.0151	2.5681 (0.9050-7.2875)	0.0763 (1.000)
chr15:89319006:C:T (p.Thr1066=)	MDE	T	0.0121	0.5753 (0.2564-1.2908)	0.1800 (1.000)
	SAS		0.0135	0.8009 (0.2961-2.1660)	0.6618 (1.000)
chr15:89320789:G:A	AAC	A	0.0781	1.2915 (0.9221-1.8087)	0.1367 (1.000)

(p.Tyr986=)	AFR		0.1069	0.9708 (0.8602-1.0956)	0.6312 (1.000)
chr15:89323415:G:A (p.Leu752=)	EUR	A	0.0115	1.1808 (1.0062-1.3856)	**0.0418** (1.000)
	MDE		0.0145	1.3753 (0.6841-2.7645)	0.3711 (1.000)
chr15:89323863:G:T (p.Ala703=)	AAC	T	0.0313	0.8464 (0.4831-1.4827)	0.5598 (1.000)
	AFR		0.0414	1.0800 (0.8925-1.3068)	0.4290 (1.000)
chr15:89324193:C:T (p.Glu662Lys)	AMR	T	0.0876	1.2189 (0.9741-1.5253)	0.0835 (1.000)
chr15:89326688:G:A (p.Arg546Cys)	AAC	A	0.0168	0.9841 (0.4940-1.9605)	0.9636 (1.000)
	AFR		0.0203	0.9364 (0.7126-1.2306)	0.6374 (1.000)
chr15:89328729:G:A (p.Leu376=)	AAC	A	0.0110	0.6223 (0.2361-1.6406)	0.3376 (1.000)
	AFR		0.0156	1.1223 (0.8380-1.5030)	0.4388 (1.000)
chr15:89329018:C:T (p.Lys316=)	CAS	T	0.0250	1.1062 (0.6696-1.8275)	0.6935 (1.000)
	EAS		0.0111	0.7910 (0.5099-1.2273)	0.2955 (1.000)
chr15:89330084:G:A (p.Ile284=)	AFR	A	0.0167	1.2636 (0.9446-1.6904)	0.1150 (1.000)
chr15:89330133:C:G (p.Gly268Ala)	MDE	G	0.0139	0.9213 (0.4371-1.9419)	0.8295 (1.000)
chr15:89333621:T:C (p.Gln45Arg)	AFR	C	0.0149	1.2505 (0.9172-1.7047)	0.1575 (1.000)
chr15:89333627:T:C (p.Gln43Arg)	AAC	C	0.0409	0.8076 (0.4999-1.3046)	0.3825 (1.000)
	AFR		0.0554	1.0339 (0.8735-1.2238)	0.6986 (1.000)

A1 = Effect allele, AAC = African Admixed, AFR = African, AJ = Ashkenazi Jewish, AMR = Latinos and Indigenous People of the Americas, CAH = Complex Admixture History, CAS = Central Asian, CES = Clinical exome sequencing, CI = Confidence interval, EAS = East Asian, EUR = European, Freq = Frequency, MDE = Middle Eastern, OR = Odds ratio, SAS = South Asian.
